# Spiritual care competencies among nursing students in the middle East and Asia: a systematic review

**DOI:** 10.1186/s12912-025-03047-3

**Published:** 2025-04-10

**Authors:** Inggriane Puspita Dewi, Hartiah Haroen, Hana Rizmadewi Agustina, Tuti Pahria, Nita Arisanti, Preeya Keawpimon

**Affiliations:** 1 Doctoral Nursing Student, Faculty of Medicine, Padjadajaran University, Bandung, West Java Indonesia; 2 Nursing Department, Faculty of Health Sciences, Universitas’ Aisyiyah, Bandung, West Java Indonesia; 3 Nursing Department, Faculty of Nursing, Padjadjaran University, Bandung, West Java Indonesia; 4 Public Health Department, Faculty of Medicine, Padjadajaran University, Bandung, West Java Indonesia; 5 Department of Nursing, Faculty of Nursing, Prince of Songkhla University, Pattani Campus, Thailand; 6 Faculty of Medicine, Padjadajaran University, Jl. Ir. Soekarno KM 21, Hegarmanah, Jatinangor, Sumedang, West Java 45363 Indonesia

## Abstract

Spiritual care is recognized as a crucial component of holistic nursing education, significantly enhancing patient well-being, health outcomes, and decision-making. However, a systematic evaluation of spiritual care education programs in the Middle East and Asia remains limited. This systematic review aims to assess the effectiveness of spiritual care education programs in enhancing undergraduate nursing students’ competencies in these regions. Following PRISMA-P guidelines and registered on PROSPERO (CRD42024552137), the study employed the PICOS framework for selection criteria: Participants (undergraduate nursing students), Intervention (spiritual nursing education programs), Comparison (education without spiritual content), Outcomes (spiritual care competencies), and Study design (RCT or quasi-experiment). Searches were conducted in Cochrane, Medline, PubMed, Sage, and Scopus from April to May 2024. Multiple reviewers independently performed data extraction and quality assessment using ROB 2 and ROBIN-I tools. A narrative synthesis approach explored data relationships and theoretical applications. Out of 1,350 reviewed articles, 10 studies involving 749 nursing students from Iran, Taiwan, and Turkey met the inclusion criteria. The interventions spanned from one month to a full semester and combined theoretical learning with clinical practice. The findings indicate that education in spiritual care significantly enhances students’ self-awareness, knowledge, attitudes, and practical skills. The study emphasizes the importance of incorporating spirituality into nursing curricula to promote a holistic approach to patient care. Limitations of the study include differences in study designs, selection bias, variability among participants, and reliance on self-reported data. Future research should aim for longitudinal studies to evaluate long-term effects and create culturally sensitive assessment tools for measuring spiritual competencies.

**Supplementary Information:**

The online version contains supplementary material available at 10.1186/s12912-025-03047-3.

## Introduction

Spirituality is fundamental to holistic, patient-centered care, providing individuals with meaning, direction, and a connection to what is considered sacred. It extends beyond religious beliefs, encompassing personal values and a deeper sense of purpose in life. While religion refers to an organized system of beliefs and practices, spirituality is a broader concept that may or may not involve religious affiliation [1–4]. In healthcare, particularly nursing, integrating spiritual care enhances patient well-being, decision-making, and coping strategies. Many individuals turn to their faith during challenging times, especially when facing serious illness. Recognizing faith as a source of support helps healthcare providers identify patients’ spiritual needs and address them appropriately [5, 6]. By incorporating spiritual care into nursing education, professionals develop the competencies to provide holistic, patient-centered services. This integration ultimately improves healthcare quality by ensuring that patients’ spiritual and emotional well-being are considered alongside their physical health [2].

Holistic patient care can be achieved by providing structure to experiences, attributing meaning to them, and offering comfort, well-being, security, and a sense of belonging [7]. Spiritual care plays a fundamental role in this approach, as it addresses patients’ emotional and existential needs alongside their physical health. As an integral part of healthcare, spiritual care often requires interdisciplinary collaboration among health professionals. It is particularly important in rehabilitation, palliative care, and general practice, where patients may seek deeper meaning and support during critical moments [8, 9]. Given its significance, nurse educators are increasingly encouraged to incorporate spirituality into nursing curricula and clinical practice [10–12]. A systematic review is essential to guide this integration effectively. Understanding and embracing spirituality equips healthcare professionals and nursing students with the skills to improve patient outcomes, enhance quality of life, and support informed decision-making [13, 14].

Existing systematic reviews have identified several methodological constraints that limit the robustness of their conclusions. Paal et al. [15] highlighted the absence of control groups in research studies and the importance of examining the validity of tools utilized to evaluate spirituality. Moreover, concerns related to biases like social desirability and questionnaire fatigue were also addressed. Mthembu et al. [16] pointed out the lack of preparedness among instructors and methodological shortcomings in integrating spirituality into health sciences education. Jones et al. [17] noted the challenges in capturing the abstract nature of spirituality and the need for diverse search terms, which could lead to the omission of relevant studies. Addressing these methodological limitations is crucial to ensure the reliability and validity of future research outcomes.

Furthermore, previous systematic reviews by Paal et al. [15] Jones et al. [17], and Crozier et al. [18] have primarily focused on North America and Europe, with limited representation from Middle Eastern and Asian contexts. Since spirituality is interpreted and practiced differently across cultures, findings from Western studies may not be fully applicable to these regions. The existing regional gap in research restricts its applicability to diverse cultural and religious backgrounds. Rykkje et al. noted that most studies on spiritual education are conducted in Western nations, highlighting the need for research in non-Western contexts. Moreover, the differing definitions of spirituality across cultures complicate the interpretation of findings [12]. A systematic review focusing on Middle Eastern and Asian contexts could foster the development of culturally relevant educational interventions, ultimately enhancing nursing students’ ability to provide holistic, patient-centered care.

This systematic review aims to assess the effectiveness of spiritual care education programs in enhancing the competencies of undergraduate nursing students in the Middle East and Asia. By identifying effective educational strategies, evaluating their impact, and addressing gaps in current research, this study provides insights into improving spiritual care training in culturally diverse settings.

The findings will contribute to developing evidence-based recommendations for integrating spiritual care into nursing curricula in these regions, particularly for those working in multicultural and multi-faith communities, where understanding diverse spiritual perspectives is critical to holistic care.

## Materials and methods

### Study design

This review aims to address the following questions:

1. What are the characteristics and effectiveness of spiritual care education programs for nursing students in the Middle East and Asia?
2. How do these programs affect students’ competencies in delivering spiritual care?

A systematic review was developed in compliance with PRISMA-P guidelines and registered on PROSPERO with registration number CRD42024552137.

### Eligibility criteria

The PICOS framework was used to structure the research inquiry to identify the key terms and create an appropriate research strategy for the study’s aim.

- P: Undergraduate nursing students or nursing college students.
- I: Spiritual nursing education program.
- C: Education program without spiritual content.
- O: Spiritual care competencies (knowledge, attitude, and skill).
- S: RCT or quasi-experiment.

### Search strategies

Electronic databases were used for research, reviewing, and reference lists. Cochrane, Medline, PubMed, Sage, Directory of Open Access Journals, Science-Direct, Pro-quest, Google Scholar, and Scopus databases were queried to access the primary studies. The exploration of databases commenced in April 2024, with the final search conducted in May 2024.

The Boolean operator OR, while terms associated with distinct components were conjoined using the AND operator. Examples of keywords in Google Scholar were: ((“Spiritual Nursing” OR “Spiritual Care in Nursing” OR “Spirituality in Nursing” OR “Spiritual Health in Nursing”) AND (“Education Program” OR “Educational Program” OR “Training Program” OR “Instructional Program” OR “Curriculum” OR “Educational Intervention”) AND (“Nursing Students” OR “Student Nurses” OR “Nursing Undergraduates” OR “Nursing Trainees” OR “Nursing Education” OR “Nursing Learners”)) AND (“spiritual care competencies”)). The detailed search methodology for exploring the keywords in databases is delineated in Appendix 1.

Rayyan Software was used to aggregate, organize, and compare retrieved papers. A team member independently did initial exploration and screening tasks, assessing titles and abstracts and eliminating duplicates. Two team members evaluated the eligibility of full texts of relevant papers (IPD and HH). Disagreements are resolved through discussion or with the assistance of another reviewer (TP, HR, NA, PK).

### Data extraction and analysis

The data extracted independently by three reviewers (IPD, HR, and PK) are depicted in authors, years, participants’ characteristics, location of study, study design, sample size, intervention, and outcomes. Data items were collected in an Excel spreadsheet. Any disagreements with the reviewer are resolved through discussion or with the help of an additional reviewer.

A third reviewer was selected based on expertise in nursing education and systematic review methodologies to ensure a rigorous review process. This reviewer was crucial in resolving discrepancies during data extraction and quality assessment. A structured reassessment process was followed, in which the third reviewer independently evaluated any inconsistencies in study inclusion, risk of bias assessment, and data synthesis. This approach helped to minimize subjectivity and enhance the reliability of the findings.

The population under study is comprised of students enrolled in an academic undergraduate nursing program. The intervention is a spiritual care learning program course or training. The educational content encompasses spirituality, spiritual nursing, personal awareness, spiritual suffering, communication skills, comparative religious studies, and the ethics of spiritual nursing. Instructional methods include lectures, online learning, simulations, role-playing, videos, group discussions, and practical exercises. The comparison is education without any content of spirituality.

The participants demonstrated improved competencies in spiritual care, including increased self-awareness, knowledge, attitudes, and practical skills, leading to better integration of spirituality in clinical practice. Studies using randomized control trials and quasi-experimental methods.

Three reviewers (IPD, HR, PK) independently evaluated the study quality using specific tools. The ROB 2 tool was utilized to conduct randomized control trials. Overall bias judgment can be low, high, or with some concern [19].

Quasi-experimental studies were evaluated using the ROBIN-I Tool. Bias judgment can be critical, serious, moderate, or low-risk [20]. The studies for the systematic review were analyzed through a narrative synthesis. Investigations on nursing sciences education regarding spirituality and spiritual care were explained.

The reviews included learning objectives, content, topic knowledge, teaching methodologies related to spirituality and spiritual care, and research summaries by country. The narrative synthesis process comprises four primary components: developing a theoretical framework, conducting an initial synthesis, examining data connections, and evaluating the robustness of the synthesis. The evaluation focuses on data that supports inferences about effects across various contexts and demographics [18].

## Results

### Characteristics of included studies

**Fig. 1:**
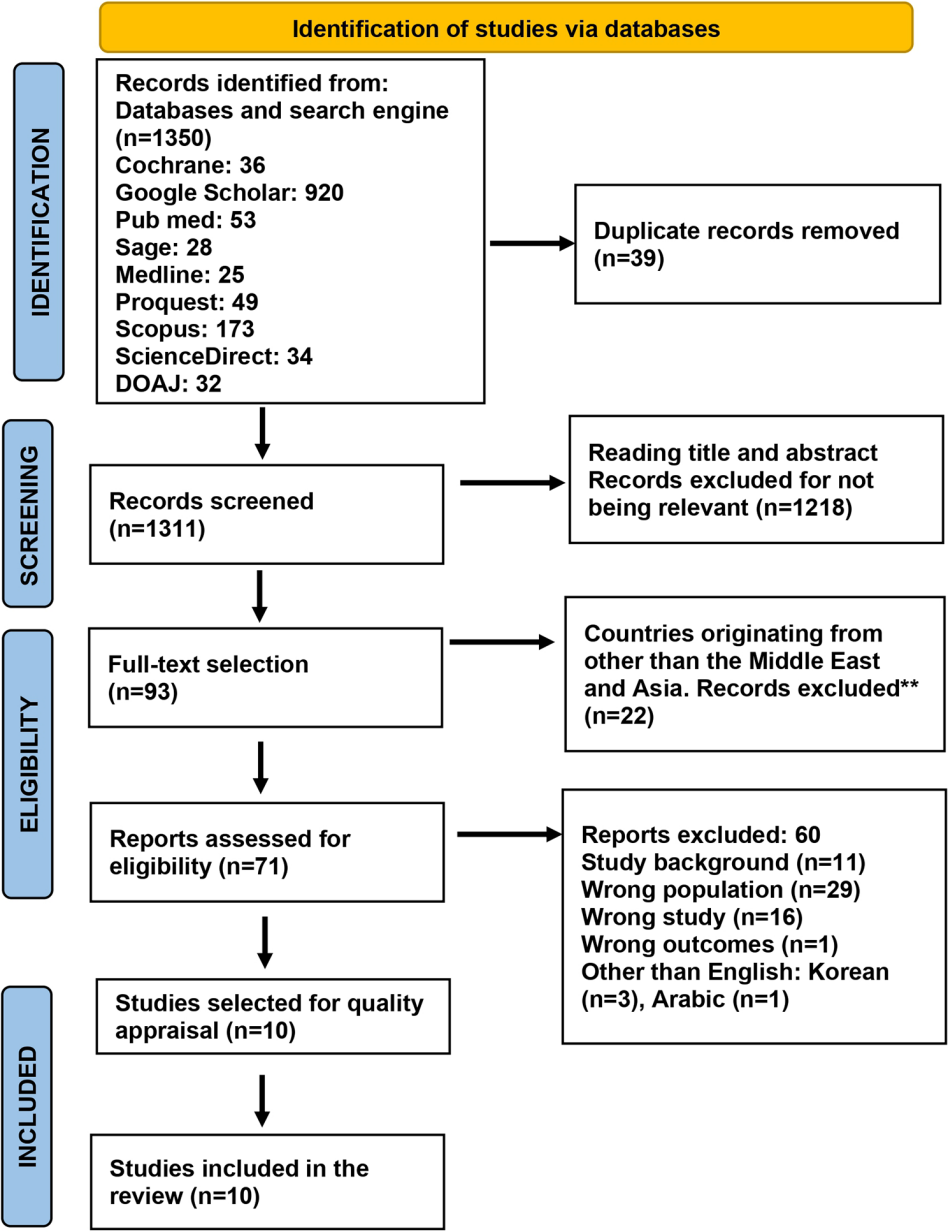
Prisma flow diagram

1350 articles were recorded from databases and search engines, with duplicate records removed from 39 articles. 1218 articles were removed for not being relevant, and 93 full-text were selected for country of origin. 22 articles came from other than the Middle East and Asia, so the selection of articles for eligibility was 71. Ten studies were included in this systematic review. Search results are recorded in a PRISMA flowchart (Fig. 1).

Table 1 presents the attributes of the studies included in our analysis. The origin countries of the studies are Iran [22–25] and Turkey [26–28]. Two study designs for RCT [24, 25] and eight quasi-experiments [22, 23, 26–31]. The studies involved 749 participants, with sample sizes ranging from 30 to 72 nursing students in each study. All participants were nursing students in their third to final semester of undergraduate university courses.

**Table 1:** Summary of findings in included studies

Study	Learning outcomes	Contents of course/training/education	Learning/teaching methods	Tools	Result and suggestion
Chiang et al. (2020), Taiwan [29]	1) Spiritual care competencies 2) Caring behavior 3) Professional Commitment 4) Religious belief	The *Spirituality and Spiritual Care* course explored connections with higher beings, nature, and interpersonal relationships. It covered spirituality, spiritual tension, needs assessment, and nursing care models. Clinical cases included spinal cord injury, terminal cancer, and relapsing depression. Self-relationships focused on mindfulness and stress perception. Interpersonal relationships were examined through Confucianism, emphasizing professionalism in nursing. Higher-being relationships explored Taiwanese religions, death interpretations, and belief-based coping, highlighted by pediatric cancer. Taoism’s perspective on human-nature connections emphasized sensory enhancement for well-being, with stroke rehabilitation as a case study.	These topics were examined using various teaching methods, such as traditional lectures, mindfulness-based stress reduction, case studies, experiential practice, and self-reflection. These topics were examined using various teaching methods, such as traditional lectures, mindfulness-based stress reduction, case studies, experiential practice, and self-reflection.	Spiritual care was assessed using the 15-item SCAS, measuring attitudes toward spiritual growth, core spirituality concepts, and spiritual nursing (Cronbach’s α = 0.95). Caring behavior was evaluated with a 28-item scale covering illness trajectory support, patient advocacy, and patient knowledge (α = 0.95). Professional commitment in nursing was measured by a 19-item scale assessing compliance, involvement, and retention (α = 0.93). The *Spiritual Health Scale (short version)* examined nursing students’ well-being, including connection, meaning, and self-awareness (α = 0.92). A 17-item *Religious Belief Scale* assessed religious effects, divinity, queries, and stress (α = 0.87)	The spiritual education course group improved more in spiritual attitudes and caring behaviors than the control group at all assessment points (*p* < 0.001). At follow-up, they also demonstrated higher professional commitment (*p* < 0.001). After a 3-week clinical practicum, the intervention group reported significantly lower stress levels (*p* < 0.001). These findings suggest that the course effectively enhanced nursing students’ spiritual attitudes, caring behaviors, and spiritual health while reducing practicum-related stress.
Frouzandeh et al. (2015), Iran [23]	Selfefficacy in providing spiritual care	The spiritual care training comprised three stages over four 2-hour sessions. The first stage covered spirituality concepts, spiritual tension, patient needs assessment, stress theories, and nursing processes. The second stage involved bedside training, holistic assessment, and care plan development. The third stage focused on small group discussions, care plan preparation, and a self-efficacy questionnaire. A follow-up post-test reassessed self-efficacy improvements after the intervention	Learning methods included speech, heuristic techniques like brainstorming, analysis of spiritual stress scenarios, small group discussions, sharing personal and clinical experiences, and deep-thinking exercises. Learning methods included speech, heuristic techniques like brainstorming, analysis of spiritual stress scenarios, small group discussions, sharing personal and clinical experiences, and deep-thinking exercises.	The questionnaire included eight statements rated on a Likert scale from none to completely (0 to 32). Content validity and surveying were used to establish its validity with 15 nursing faculty members. Reliability was assessed with a Cronbach’s alpha coefficient of 81%.	Nursing students’ self-efficacy in providing spiritual care increased from 13.74 (pretest) to 21.1 (post-test, *p* < 0.001). The significant improvement highlights the effectiveness of structured training. Findings suggest that a specialized curriculum enhances students’ ability to understand spirituality, assess patients’ spiritual needs, and develop appropriate care plans.
Hsiao et al. (2012), Taiwan [30]	1) Spiritual health status 2) Level of clinical practice stress	The initial phase of the *Spiritual Life Program (SLP)*, based on Lazarus and Folkman’s stress-coping theory, included two modules on personal spiritual awareness and spirituality. It then focused on strategies for enhancing spiritual well-being through stress relief, relationships, gratitude, self-worth, and purposeful living, fostering reflection and spiritual growth	The learning strategies included lectures on spirituality, reflective sheets with questions to explore personal meaning, discussions for sharing spiritual experiences, and practical activities like meditation to reinforce learning and self-awareness. These methods aimed to deepen understanding and personal growth through reflection, dialogue, and experiential practice.	The Spiritual Health Scale (SHS) uses qualitative and quantitative methods to assess spiritual well-being. It consists of 47 items across five subcategories, with reliability (Cronbach’s alpha: 0.77–0.89). The Clinical Practice Stress (CPS) scale measures nursing students’ stress during practice using six subscales (29 items, α = 0.91)1)	The Spiritual Learning Program (SLP) significantly improved spiritual well-being in the experimental group (*p* < 0.001), though the effect was temporary. The experimental group showed a greater reduction in Clinical Practice Stress (CPS) scores than the control group (*p* < 0.05, t = 3.771, *p* < 0.001). A longitudinal study is needed to assess long-term changes in spiritual well-being.
Ekramifar et al. (2018), Iran [22]	Moral sensitivity status	The education covers spiritual characteristics, skills, and well-being, emphasizing spirituality in education and nursing. It explores self-awareness, spirituality’s link to health, and its role in mental health, including spiritual problem-solving, forgiveness, faith, patience, meditation, and generosity, along with their stages, consequences, and personal growth	The intervention methods included lectures, question-answer sessions and in clinical setting.	The Moral Sensitivity Questionnaire (K-MSQ) assesses moral sensitivity using 30 questions across five dimensions: patient-centered care, professional accountability, ethical dilemmas, ethical decision-making, and benevolence. Originally developed in Sweden, its reliability was tested through a test-retest analysis, yielding a coefficient of 0.89 in this study	The intervention group showed a significantly higher mean moral sensitivity score than the control group (*P* < 0.001). Future studies should examine how demographic factors influence moral sensitivity in nursing students and explore the impact of training methods, such as workshops, on their moral sensitivity.
Karaca et al. (2024), Turki [26]	1) Spiritual care competencies 2) Spiritual care perception	The curriculum included four hours each on spiritual care concepts, spiritual needs in palliative care, and the significance of spiritual care, plus six hours on nursing diagnosis and interventions. Case studies emphasized critical thinking. Three experts in psychiatry, fundamentals, and medical-surgical nursing evaluated the program content	The course was delivered through traditional face-to-face instruction, incorporating theoretical presentations, collaborative activities, idea generation, and interactive question-and-answer methodologies	The Spiritual Support Perception Scale (SSPS) measures perceptions of moral support and spiritual care, with higher scores indicating stronger perceptions. The scale has a maximum score of 60 and a Cronbach’s alpha of 0.82. The Turkish version of the Spiritual Care Competence Scale (SCCS) assesses nursing proficiency in spiritual care across three domains. It consists of 27 items and has a reliability coefficient (Cronbach’s alpha) of 0.90.	The intervention group showed significantly higher mean scores than the control group in personal support and patient counseling (*p* < 0.002), attitude toward patient spirituality and communication (*p* < 0.004), and the spiritual support scale (*p* < 0.003). Expanding this study to include spiritual care coursework for junior and senior students is recommended to compare cohorts and assess their knowledge levels
Momennasab et al. (2019), Iran [24]	1) Spiritual well-being 2) Attitude toward spiritual care	The scenarios covered themes such as meaning and purpose, relationships with God and others, forgiveness, prayer, rituals, hope, and the presence of family and nurses. Developed using nursing textbooks and clinical observations, the scenarios were revised and validated by five nursing faculty members	Kolb’s experiential learning emphasizes learning through experience. Group reflections followed Gibbs’ reflective cycle’s six stages: describing events, expressing feelings, evaluating, analyzing, concluding, and planning actions. This structured approach deepened understanding and improved learning through critical thinking and self-assessment	The Spiritual Well-Being Scale (SWBS) assessed participants’ spiritual well-being through 20 questions on a 6-point Likert scale, with a Cronbach’s alpha of 0.82. The Spirituality & Spiritual Care Rating Scale (SSCRS) measured attitudes on spirituality and care, with reliability scores between 0.64 and 0.84	The mean score differences pre-post in intervention group: 1) Spiritual well being (*p* = 0,0003) 2) Attitude to spiritual care (*p* = 0,047) No significance found between group
Özveren et al. (2019), Turki [27]	To achieve Spiritual care competencies	The intervention covered pain management, symptom control, loss, mourning, death, postmortem care, spirituality, spiritual care, influencing factors, the nursing process in spiritual care, spiritual distress as a diagnosis, patient-centered spiritual approaches, and the nurse’s role in family support.	No information	The SSCR, developed by McSherry (2002), measures spirituality and spiritual care with 17 questions. Its Cronbach’s alpha was 0.76, and in this study, it was 0.74, confirming reliability. The scale evaluates various dimensions related to spirituality and spiritual care.	Student nurses’ scores significantly improved post-training (*p* < 0.001), indicating enhanced perceptions of spirituality and spiritual support. The study recommends integrating more spiritual care training into nursing education to strengthen students’ understanding and application of spiritual care in practice
Sharifi et al. (2024), Iran [25]	To achieve Spiritual care competencies	The intervention featured an empowering program covering spirituality, spiritual care, assessment, implementation, communication skills, dignity, individual support, counseling, hope therapy, and quality of life improvement, enhancing nurses’ ability to provide comprehensive spiritual care	The content of the intervention was explained and taught in the form of lectures and small group discussions	The validity and reliability of the SCCS were confirmed in Iran. Nasehi et al. (2013) found a Cronbach’s alpha of 0.78, while another study reported an alpha of 0.77 for the entire instrument, with subcategory values ranging from 0.65 to 0.85.	Significant differences were found between groups in overall spiritual care competence (*p* < 0.037) and two subscales: assessment and implementation of spiritual care, and professionalism in spiritual care (*p* < 0.05). Enhancing spiritual care skills in nursing students improves patient care, highlighting the need for greater curricular emphasi
Tsai et al. (2019), Taiwan [31]	To achieve meaning of life, positive beliefs, and well-being among nursing students	A life-education intervention. The fundamental beliefs in life include self-awareness, determination, cognitive processes, introspection, broadening of horizons, independence, self-knowledge, personal encounters, and purpose in life	Simulated directed learning was used, integrating YouTube videos, e-books, and online movies. Nursing students accessed three topic contents in class and could download materials from an e-learning platform for future reference, enhancing flexibility and retention in their learning process	The study utilized instruments from the Life Attitude Profile by [36] and the Positive Coping, Spirituality and Well-being Scale by [37]. The content validity index (CVI) of the questionnaire was established at 0.95 by 7 expert scholars.	Significant differences were found in meaning of life, positive belief, and well-being between the intervention and control groups (*p* < 0.001). Directed learning simulations in life education interventions enhance these aspects, emphasizing the need for their inclusion in nursing education
Yilmaz et al. (2014), Turki [28]	To achieve Spirituality and Spiritual Care competencies	1) The scenarios included patients with cancer or requiring heart surgery, aiming to challenge personal systems of meaning and purpose in life. 2) Concepts were discussed with patient care plans and	Students applied these concepts in clinical settings during a year-round internship with two semesters of 15 weeks each	The Spirituality and Spiritual Care Rating Scale by [34] The Cronbach’s alpha, on the Turkish version of the scale, was 0.76	Senior-level nursing students show significant differences in spiritual knowledge and attitudes (*p* < 0.001). Integrating interactive methods and spirituality into the curriculum enhances these aspects. Gordon’s FHPs provide a holistic framework for incorporating spirituality into nursing education effectively

Intervention in spiritual education was provided for varying durations, ranging from one month to a whole semester (16 weeks), covering materials, content, and learning outcomes. Only four of the ten studies integrated theoretical learning with clinical practice in hospitals [23, 25, 29, 30] while the rest solely took place in a classroom setting. One study described incorporating Buddhist religious beliefs into spiritual courses [29] while the other studies did not explicitly address the integration of the predominant religion in the area.

Spiritual education intervention was compared with face-to-face lessons or regular education without spiritual content in eight studies using a traditional course model. Two studies did not have a control group for comparison.

All ten studies assess the competencies encompassing attitudes, knowledge, and abilities resulting from spiritual care education. The outcomes evaluated include spiritual care abilities, self-efficacy, moral sensitivity, attitudes toward spiritual care, caring actions, and spiritual health. Additionally, the research examines students’ perceived support for spiritual care, commitment to the profession, and spiritual well-being. All studies reported significant values ranging from less than 0.001 to 0.004, indicating that spiritual care instruction positively impacted the assessed outcomes.

### Quality assessment

**Fig. 2:**
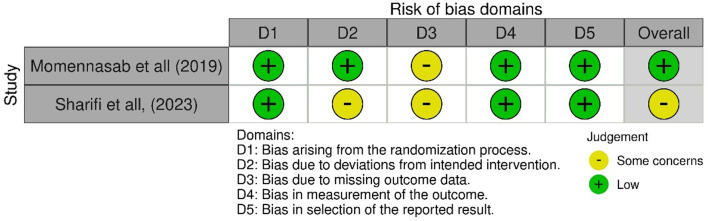
Risk of bias randomized control trial

The primary risk of biased findings from RCT studies indicates some concern about bias due to missing outcome data in the studies by Momennasab et al. [24] and Sharifi et al. [25]. Additionally, participants in the survey by Sharifi et al. were not blinded to treatment assignment, as they were aware of whether they were participating in group reflection sessions or attending a lecture, refer to Fig. 2.

**Fig. 3:**
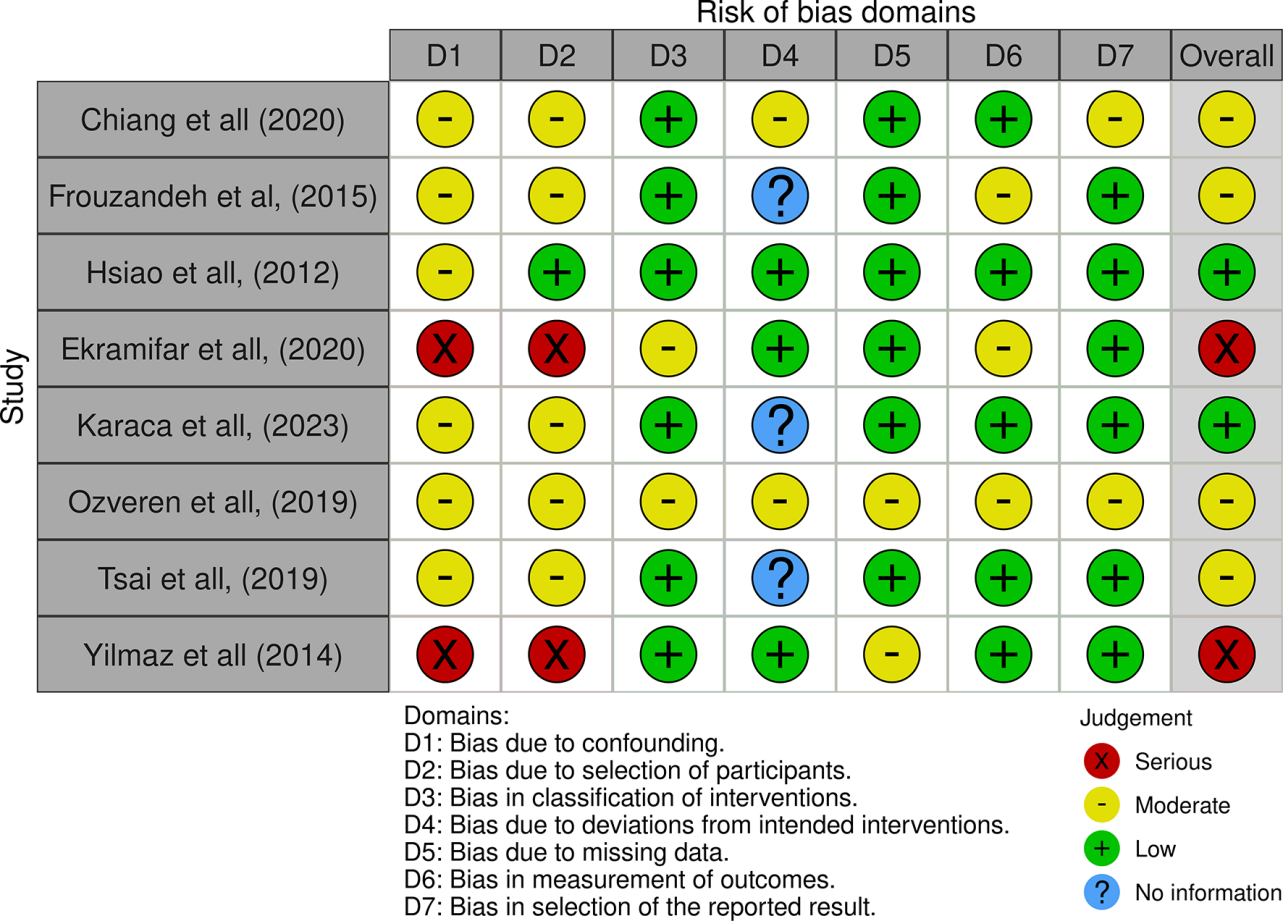
Risk of bias quasi-experimental

Eight research articles were assessed for bias risk using seven categories: confounding bias, participant selection, intervention classification, deviations from intended interventions, missing data, outcome measurement, and selection of reported results. The articles were evaluated and rated as critical, serious, moderate, low, or without information, refer to Fig. 3.

The findings revealed a varying degree of bias among the studies. Three articles exhibited a serious risk of bias overall, highlighting significant concerns in multiple categories. Specifically, studies by Chiang et al. [29], Frouzandeh et al. [23], and Hsiao et al. [30] each had serious biases, mainly due to confounding and selection of participants. Another study by Özveren et al. [27] also showed a serious overall risk, with moderate biases consistently across most categories.

Studies by Ekramifar et al. [22], and Yilmaz et al. [28] determined a critical risk of bias, the highest concern level. The study was critically biased due to confounding, while other studies had critical and serious biases, indicating substantial methodological flaws that could compromise the validity of their findings. Two articles demonstrated a moderate risk of bias. Although the study by Karaca et al. [26] lacked some information with some moderate biases, Tsai et al. [31] was more robust than the others but still had limitations that needed careful consideration. Overall, the analysis highlights that most articles have notable biases that could impact their reliability, emphasizing the need for a cautious interpretation of their findings.

### Synthesis of the results

**Fig. 4:**
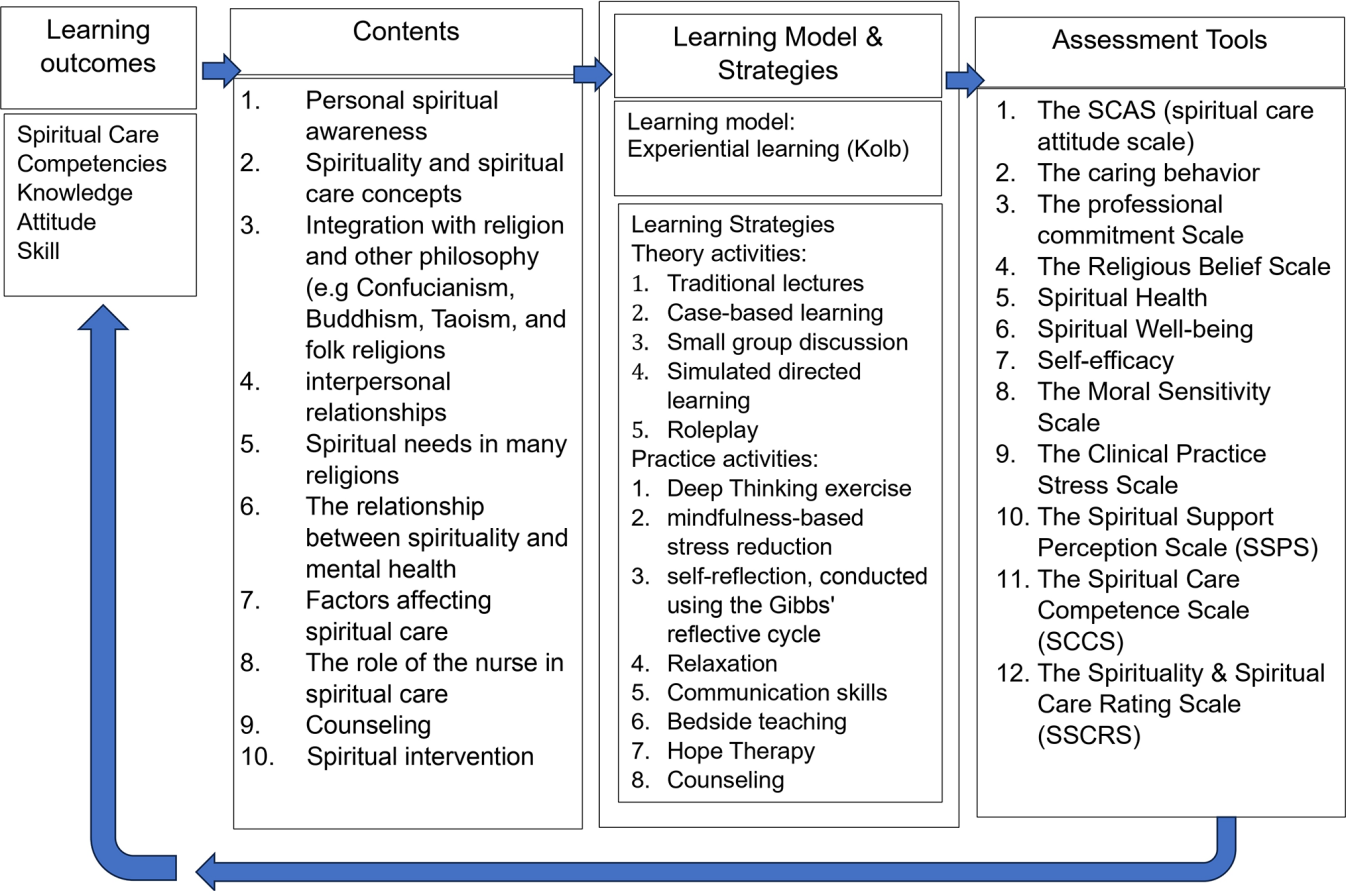
Linking learning outcomes, assessment tools and teaching/learning activities in spiritual care education

Narrative synthesis allows for describing patterns, exploring data relationships, and applying theory to the findings. It involves referencing original studies when summarizing and explaining the findings to enhance credibility. Spiritual education programs are developed by defining learning outcomes and creating study materials to achieve these competencies. It is essential to choose appropriate learning strategies, as illustrated in Fig. 4.

The diagram presents a comprehensive framework for spiritual care education, encompassing learning outcomes, assessment tools, learning models, and teaching strategies. The theoretical framework integrates knowledge, attitudes, and skills necessary for spiritual care, forming the foundation for educational objectives. A preliminary synthesis highlights diverse assessment tools, such as scales for attitudes, behaviors, professional commitment, and spiritual health, ensuring a thorough evaluation of competencies.

Investigating data connections reveals that theoretical and practical activities are intertwined to enhance learning. Theoretical methods include traditional lectures, experiential learning, and case-based discussions, while practical activities emphasize mindfulness, self-reflection, and bedside teaching, ensuring a balanced approach. These connections demonstrate how theoretical knowledge is applied in practical settings, reinforcing learning.

Assessing the robustness of this synthesis involves evaluating the evidence supporting the framework’s effectiveness across various populations and contexts. The comprehensive nature of the framework, covering multiple dimensions of spiritual care, suggests it is adaptable and applicable in diverse healthcare settings, thereby enhancing the overall quality of spiritual care education.

## Discussion

### Effectiveness of spiritual care education

Spiritual care programs significantly improved nursing students’ competencies, particularly in developing self-awareness, empathy, and communication skills, as shown in Table 1. The reviewed studies indicate that students who received structured spiritual education demonstrated a better understanding and implementation of spiritual care in clinical settings [15, 17]. As Figure Four illustrates, combining theoretical knowledge with practical application is crucial for enhancing learning outcomes. Students engaged in experiential learning activities—such as case studies, role-playing, and clinical exposure—reported greater confidence and competence in addressing patients’ spiritual needs [10, 12, 32]. The Spiritual Care Competence Scale (SCCS) and the Spirituality & Spiritual Care Rating Scale (SSCRS) are frequently used to assess outcomes related to spiritual care competence, indicating their reliability and validity in educational settings [33].

Additionally, research suggests that incorporating reflection-based learning, such as Gibbs’ Reflective Cycle, can further enhance students’ ability to integrate spirituality into nursing practice [24]. This structured approach allows students to critically assess their experiences and better understand patients’ spiritual needs [25]. Experiential learning through direct patient interaction is emphasized as a crucial component, allowing students to apply spiritual care theories in real-world settings, which leads to greater confidence in their skills [30].

### Cultural and contextual considerations

The effectiveness of spiritual care education is shaped by cultural and religious contexts. In Taiwan, programs incorporated Buddhist teachings [29], while those in Iran and Turkey were aligned with Islamic principles [26, 27]. The remaining seven studies on spiritual care learning content did not affiliate with any specific religion. Understanding these variations is essential for designing culturally relevant spiritual education programs. The findings indicate that educators should modify course content to align with local beliefs, ensuring that students can deliver patient-centered spiritual care in their respective environments.

While religious teachings often inform spiritual care education, it is important to distinguish between spirituality and religion. Spiritual care addresses patients’ existential needs, values, and sources of meaning, whereas religious care involves faith-specific practices, rituals, and doctrines. These variations emphasize the importance of culturally relevant education that aligns with local beliefs and practices. Differences in curriculum structure were also observed, with some programs integrating spirituality into broader nursing education while others offered it as a standalone module. Findings suggest that structured and standardized spiritual care curricula lead to better competency outcomes, underscoring the need for consistency in educational frameworks [11, 16].

The review also emphasizes the differences in the structure of spiritual care education across various countries. Some programs incorporated spirituality into broader nursing curricula, while others offered it as a separate module. In countries with a more organized approach to spiritual care education, students demonstrated greater improvements in competency. This highlights the necessity for standardizing curricula in this area [16, 34]. Incorporating interfaith perspectives in spiritual care training is beneficial, as it enables students to develop an inclusive approach when interacting with patients from diverse religious backgrounds [35].

### Study bias and quality assessment

The reliability of findings in this review is influenced by study biases, including selection bias, lack of blinding, and missing data. Selection bias was evident in studies that did not use random sampling, which may limit the generalizability of results. The lack of blinding in several studies, where participants were aware of their intervention status, could introduce response bias, leading to inflated self-reported improvements in spiritual care competencies [24, 25]. Additionally, missing data in follow-up assessments raises concerns about the long-term sustainability of observed effects [17].

Despite these limitations, studies that employed rigorous methodological approaches, such as randomized controlled trials and validated assessment tools, provide valuable insights into the effectiveness of spiritual care education [31, 35]. Future research should aim to mitigate bias by incorporating double-blind designs, ensuring complete follow-up data, and using mixed-methods approaches to triangulate findings. Recognizing these biases allows for a balanced interpretation of the results, acknowledging both the strengths of well-designed studies and the limitations posed by methodological weaknesses.

### Limitations

A key challenge in synthesizing findings from different studies is the significant variability in study designs, intervention durations, and assessment tools. Many studies relied on self-reported measures, introducing potential bias. Moreover, only a few studies incorporated long-term follow-ups to assess the sustainability of competency gains over time. Variability in assessment tools used across different studies also complicates cross-study comparisons and reduces the generalizability of findings.

The study has limitations, as it only includes articles from three countries in the target region: Iran, Turkey, and Taiwan. This scarcity may affect the generalizability of the findings. The limited representation could be due to variations in research focus, the availability of studies, and the emphasis on spiritual care education within those institutions across the region.

### Implication for nursing education

The findings emphasize the importance of integrating spiritual care training into nursing curricula. Educators should utilize evidence-based strategies such as experiential learning, role-playing, and interdisciplinary collaborations. Furthermore, developing culturally sensitive teaching models can enhance the effectiveness of this training. By incorporating spiritual care education into core nursing programs, institutions can better prepare students to deliver holistic, patient-centered care.

Additionally, the integration of spiritual care should extend beyond theoretical instruction and be reinforced through clinical practice and mentorship programs. In our increasingly globalized world, equipping nurses to provide culturally and spiritually competent care is essential for improving patient outcomes and ensuring comprehensive healthcare practices.

### Future directions

Future research should prioritize the development of standardized, culturally sensitive assessment tools to accurately measure competencies in spiritual care. Additionally, longitudinal studies are necessary to evaluate the long-term effects of spiritual care education on nursing students’ professional development.

Collaborative research efforts among institutions in the Middle East and Asia could further enhance the generalizability of findings and contribute to the creation of robust educational frameworks. More research should also explore the role of technology in spiritual care education, particularly in e-learning and simulation-based training, to expand accessibility and enhance learning experiences.

## Conclusion

This review demonstrates that spiritual care education significantly enhances nursing students’ competencies in the Middle East and Asia. However, variability in study design, reliance on self-reported data, and limited regional representation present challenges to broader applicability. Future research should focus on refining methodologies, expanding cultural considerations, and standardizing assessment frameworks.

## Electronic supplementary material

Below is the link to the electronic supplementary material.

Supplementary Material 1

## Data Availability

No datasets were generated or analysed during the current study.

## Abbreviations

PICOS: Population, Intervention, Comparison, Outcome, and Study Design
PRISMA-P: Preferred Reporting Items for Systematic Review and Meta-Analysis Protocols
PROSPERO: International Prospective Register of Systematic Reviews
SCCS: The Spiritual Care Competence Scale
SSCRS: The Spirituality & Spiritual Care Rating Scale
RCT: Randomized Control Trial
ROB 2: Risk of Bias 2 Tool (for randomized trials)
ROBINS-I: Risk of Bias In Non-randomized Studies - of Interventions
QE: Quasi-Experimental

## References

[1] Murgia C, Notarnicola I, Caruso R, De Maria M, Rocco G, Stievano A. Spirituality and religious diversity in nursing: A scoping review. MDPI. Sep. 01, 2022. 10.3390/healthcare10091661. 10.3390/healthcare10091661 PMC9498726 36141273

[2] Klimasiński M, et al. Spiritual distress and spiritual needs of chronically ill patients in Poland: A cross-sectional study. Int J Environ Res Public Health. May 2022;19(9). 10.3390/ijerph19095512. 10.3390/ijerph19095512 PMC9101665 35564907

[3] Brian P, Hughes R, Elk C, DeGregory D, Graham EJ, Hal, Ressallat J. Spiritual​ care​ and​ nursing:​ A ​nurse’s​contribution​​ and practice. New York: Spiritual Care Association; 2017. [Online]. Available: https://www.healthcarechaplaincy.org/

[4] Soriano G, Aranas FC, Tejada RS. Caring behaviors, spiritual, and cultural competencies: a holistic approach to nursing care. Bedan Res J. 2019;4. https://bedanjournal.org/index.php/berj/article/view/5

[5] Murgia C, Notarnicola I, Rocco G, Stievano A. Spirituality in nursing: A concept analysis, Nurs Ethics. Aug. 2020;27(5):1327–1343. 10.1177/0969733020909534 10.1177/0969733020909534 32281485

[6] Galutira GD, Valenzuela JP, Basatan C, Palaganas EC. Spirituality and spiritual care in nursing: A literature review, Philippine Journal of Nursing. 2019;1(89). [Online]. Available: https://www.researchgate.net/publication/343336490

[7] World Health Organization. WHOQOL and Spirituality, Religiousness and Personal Beliefs (SRPB). Geneva, Switzerland: World Health Organization, 1998. Accessed: Dec. 05, 2024. [Online]. Available: https://apps.who.int/iris/handle/10665/70897

[8] Andersen AH, Assing Hvidt E, Hvidt NC, Roessler KK. ‘Maybe we are losing sight of the human dimension’–physicians’ approaches to existential, spiritual, and religious needs among patients with chronic pain or multiple sclerosis. A qualitative interview-study, Health Psychol Behav Med. Jan. 2020;8(1):248–269. 10.1080/21642850.2020.1792308 10.1080/21642850.2020.1792308 PMC8114351 34040871

[9] Penman J. Finding paradise within: How spirituality protects palliative care clients and caregivers from depression, Journal of Holistic Nursing. Sep. 2018;36(3):243–254. 10.1177/0898010117714665 10.1177/0898010117714665 28612639

[10] Cone PH, Giske T. Integrating spiritual care into nursing education and practice: strategies utilizing open journey theory. Nurse Educ Today. Dec. 2018;71:22–5. 10.1016/j.nedt.2018.08.015. 10.1016/j.nedt.2018.08.015 30216754

[11] Chandramohan S, Bhagwan R. Utilization of spirituality and spiritual care in nursing practice in public hospitals in Kwazulu-Natal, South Africa. Religions (Basel). 2016;7(3). 10.3390/rel7030023.

[12] Rykkje L, Søvik MB, Ross L, McSherry W, Cone P, Giske T. Educational interventions and strategies for spiritual care in nursing and healthcare students and staff: A scoping review, J Clin Nurs. Jun. 2022;31(11–12):1440–1464. 10.1111/jocn.16067 10.1111/jocn.16067 34611922

[13] Susa B, Bastable. Nurse as educator principles of teaching and learning for nursing practice, 5th edition. Burlington, Massachusetts: Jones & Bartlett, 2019. Accessed: Jul. 25, 2024. [Online]. Available: https://swu.phinma.edu.ph/wp-content/uploads/2021/05/Nurse-as-Educator-Principles-of-Teaching-and-Learning-for-Nursing-Practice-by-Susan-B.-Bastable-z-lib.org_.pdf

[14] Ross L, et al. Nursing and midwifery students’ perceptions of spirituality, spiritual care, and spiritual care competency: A prospective, longitudinal, correlational European study. Nurse Educ Today. Aug. 2018;67:64–71. 10.1016/j.nedt.2018.05.002. 10.1016/j.nedt.2018.05.002 29763841

[15] Paal P, Helo Y, Frick E. Spiritual care training provided to healthcare professionals: A systematic review, Journal of Pastoral Care and Counseling. Mar. 2015;69(1):19–30. 10.1177/1542305015572955 10.1177/1542305015572955 26162203

[16] Mthembu TG, Wegner L, Roman NV. Teaching spirituality and spiritual care in health sciences education: A systematic review, African Journal for Physical Activity and Health Sciences 2016;22(1):1036–1057. [Online]. Available:http://hdl/handle.net/10520/EJC200068

[17] Jones KF, Paal P, Symons X, Best MC. The content, teaching methods and effectiveness of spiritual care training for healthcare professionals: A mixed-methods systematic review, Sep. 01, 2021, Elsevier Inc. 10.1016/j.jpainsymman.2021.03.013 10.1016/j.jpainsymman.2021.03.013 33757893

[18] Crozier D, Greene A, Schleicher M, Goldfarb J. Teaching spirituality to medical students: a systematic review. J Health Care Chaplain. 2022;28(3):378–99. 10.1080/08854726.2021.1916332. 34137668 10.1080/08854726.2021.1916332

[19] McGuinness LA, Higgins JPT. Risk-of-bias visualization (robvis): An R package and Shiny web app for visualizing risk-of-bias assessments, Res Synth Methods. n/a, no. n/a, Apr. 2020. 10.1002/jrsm.1411 10.1002/jrsm.1411 32336025

[20] Sterne JA, et al. ROBINS-I: A tool for assessing risk of bias in non-randomised studies of interventions. BMJ (Online). 2016;355. 10.1136/bmj.i4919. 10.1136/bmj.i4919 PMC5062054 27733354

[21] Dehkordi AH, Mazaheri E, Ibrahim HA, Dalvand S, Gheshlagh RG. How to write a systematic review: A narrative review. Wolters Kluwer Medknow Publications. 2021. 10.4103/ijpvm.IJPVM_60_20. 10.4103/ijpvm.IJPVM_60_20 PMC8218799 34249276

[22] Ekramifar F, Farahaninia M, Mardani Hamooleh M, Haghani H. The effect of spiritual training on the moral sensitivity of nursing students, Journal of Client-centered Nursing Care. Nov. 2018:213–222 10.32598/jccnc.4.4.213

[23] Frouzandeh N, Aein F, Noorian C. Introducing a spiritual care training course and determining its effectiveness on nursing students’ self-efficacy in providing spiritual care for the patients. J Educ Health Promot. 2015;4:34. 10.4103/2277-9531.157189. 26097848 10.4103/2277-9531.157189 PMC4456868

[24] Momennasab M, Shadfard Z, Jaberi A, Najafi SS, Hosseini FN. The effect of group reflection on nursing students’ spiritual well-being and attitude toward spiritual care: A randomized controlled trial. Invest Educ Enferm. 2019;37(1). 10.17533/udea.iee.v37n1e09. 10.17533/udea.iee.v37n1e09 PMC7871466 31083846

[25] Sharifi K, Saeidnejad Z, Hosseinian M, Aghajani M. The effect of an empowerment program on the spiritual care competence of nursing students: A randomized clinical trial study, Romanian Journal of Military Medicine. Mar. 2024;127(2):98–104. 10.55453/rjmm.2024.127.2.2

[26] Karaca T, Altınbaş Y. Spiritual care support perception and spiritual care competence of nursing students in Turkey: A quasi-experimental study, J Relig Health. Jun. 2024;63(3):1775–1785. 10.1007/s10943-023-01931-3 10.1007/s10943-023-01931-3 37847445

[27] Özveren H, Kırca K. Influence of palliative care training on last-year nursing department students’ perception on regarding spirituality and spiritual Care: A single-group pretest–posttest intervention study, J Relig Health. Jun. 2019;58(3):860–869. 10.1007/s10943-018-0701-4 10.1007/s10943-018-0701-4 30229412

[28] Yilmaz M, Gurler H. The efficacy of integrating spirituality into undergraduate nursing curricula, Nurs Ethics. Dec. 2014;21(8):929–945. 10.1177/0969733014521096 10.1177/0969733014521096 24644252

[29] Chiang YC, Lee HC, Chu TL, Han CY, Hsiao YC. A spiritual education course to enhance nursing students’ spiritual competencies. Nurse Educ Pract. 2020;49. 10.1016/j.nepr.2020.102907. 10.1016/j.nepr.2020.102907 33220574

[30] Hsiao YC, Chiang HY, Lee HC, Chen SH. The effects of a spiritual learning program on improving spiritual health and clinical practice stress among nursing students, Journal of Nursing Research. Dec. 2012;20(4):281–290. 10.1097/jnr.0b013e318273642f 10.1097/jnr.0b013e318273642f 23154439

[31] Tsai FJ, Hu YJ, Chen CY, Yeh GL, Tseng CC, Chen SC. Simulated directed-learning in life-education intervention on the meaning of life, positive beliefs, and well-being among nursing students: A Quasi-experimental study, Medicine. Jul. 2019;98(27):e16330. 10.1097/MD.0000000000016330 10.1097/MD.0000000000016330 PMC6635261 31277181

[32] Giske T et al. Apr., Developing and testing the EPICC Spiritual Care Competency Self-Assessment Tool for student nurses and midwives, J Clin Nurs. 2023;32(7–8):1148–1162. 10.1111/jocn.16261 10.1111/jocn.16261 35285563

[33] Cone PH et al. Oct., Strengths and challenges with spiritual care: Student feedback from the EPICC Spiritual Care Self-Assessment Tool, Nurs Open. 2023;10(10): 6923–6934. 10.1002/nop2.1946 10.1002/nop2.1946 PMC10495739 37475149

[34] Ross L, et al. Development of a spiritual care education matrix: factors facilitating/hindering improvement of spiritual care competency in student nurses and midwives. Nurse Educ Today. Jul. 2022;114. 10.1016/j.nedt.2022.105403. 10.1016/j.nedt.2022.105403 35597195

[35] Daghan S. Nursing students’ perceptions of spirituality and spiritual care; an example of Turkey. J Relig Health. Feb. 2018;57(1):420–30. 10.1007/s10943-017-0416-y. 10.1007/s10943-017-0416-y 28551730

[36] Ho YC. The life attitude profile. Volume 35. Taiwan: Bulletin of National Taiwan Normal University; 1990.

[37] Lin WT, Yu MN. The study of positive psychology intervention effects for promoting college students’ well-being. Taiwan: National Chen-Gchi University; 2016.

